# 568 Diagnosis and Management of Occult Heart Failure in Burn Patients Admitted with Amphetamine Intoxication

**DOI:** 10.1093/jbcr/iraf019.197

**Published:** 2025-04-01

**Authors:** Max Silverstein, Yvonne Karanas, Vina Vargas, Lea Lyn Zaballero, Clifford Sheckter

**Affiliations:** Division of Plastic and Reconstructive Surgery, Stanford University; Santa Clara Valley Medical Center; Santa Clara Valley Medical Center; Santa Clara Valley Medical Center; Santa Clara Valley Medical Center

## Abstract

**Introduction:**

10-30% of burn patients test positive for amphetamines on admission. Chronic amphetamine use is associated with heart failure, which can affect critical care and surgical approaches. Undiagnosed heart failure in an acutely burned patient can lead to morbidity in the operating room or intensive care unit. We aimed to characterize the prevalence of right and left heart failure in burn patients who use amphetamines and suggest management strategies.

**Methods:**

All patients with burns and confirmed amphetamine toxicology (cocaine or methamphetamine) were extracted from a verified burn center’s database from 2017 to mid-2024. Perioperative evaluation included transthoracic echocardiogram (TTE) for high-risk amphetamine users meeting screening criteria. The prevalence of TTE was measured for all patients with amphetamine positivity. Predictors of having an abnormal TTE were modeled with logistic regression. Ejection fraction (EF), right ventricular systolic pressure (RVSP), and diastolic function were recorded on initial TTE. Heart failure management was described in terms of perioperative management and anesthesia approach. Patient demographics, burn characteristics, and mortality were reported.

**Results:**

Of 406 amphetamine-positive patients, 127 received TTE (31%) based on screening criteria. 66% of TTEs demonstrated normal heart function. Heart failure with reduced ejection fraction was observed in 10 (8%) patients with median EF of 29%; heart failure with preserved ejection fracture was observed in 4 (3%) of patients. Diastolic dysfunction was seen in 21 (17%) patients. Pulmonary hypertension was observed in 20 (16%) patients with a median RVSP of 50 (IQR 38, 61); 3 patients had both reduced ejection fraction and pulmonary hypertension. 27 patients (21%) were considered high-risk for anesthesia based on reduced EF and/or pulmonary hypertension. The median age of this cohort was 57 years (IQR 43, 61), median TBSA was 13% (IQR 4, 25), and 78% were male. 37% (10) received cardiology consult, 30% (8) required preoperative diuresis, 11% (3) underwent surgery with regional anesthesia only, 55% (15) received wound care only, and 10% (3) died. Clinically significant findings on TTE were associated with older age (53 years vs 46 years, p=0.017) and larger TBSA (18% vs 9%, p=0.004).

**Conclusions:**

31% of all amphetamine-positive burn patients underwent TTE, of which 21% were diagnosed with heart failure with reduced EF and/or pulmonary hypertension. Perioperative optimization including diuresis and regional anesthesia were effective at mitigating risk for many patients. Mortality was high in these patients, at 10%.

**Applicability of Research to Practice:**

Burn practitioners should be aware of the incidence of left and right heart failure in amphetamine-positive patients. TTE should be obtained preoperatively to guide surgical management.

**Funding for the Study:**

The senior author is is supported by a grant from the National Institutes of Health. No specific funding was received for this study.

